# The complete chloroplast genome of a medicinal plant, *Abutilon theophrasti* Medik. (Malvaceae)

**DOI:** 10.1080/23802359.2020.1835582

**Published:** 2020-11-20

**Authors:** Junlin Yu, Weiyin Jin, Yiheng Wang, Qingjun Yuan

**Affiliations:** a School of Medicine and Pharmacy, Tonghua Normal University, Tonghua, PR China; b National Resource Center for Chinese Meteria Medica, China Academy of Chinese Medical Sciences, Beijing, PR China

**Keywords:** *Abutilon theophrasti*, chloroplast genome, phylogenetic analysis

## Abstract

*Abutilon theophrasti* Medik. is an annual weed, widely distributed in Asia and Europe. The complete chloroplast genome reported here is 160,446 bp in length, including two inverted repeats (IRs) of 25,064 bp, which are separated by a large single-copy (LSC) and a small single-copy (SSC) of 89,089 and 21,229 bp, respectively. The whole chloroplast genome of *A. theophrasti* contains 113 distinct genes, including 79 protein-coding genes, 30 transfer RNA, and four ribosome RNA. Phylogenetic analysis indicated that *A. theophrasti* is located in the basal position in Malveae.

*Abutilon theophrasti* Medik. (velvetleaf) is an annual plant native to southern Asia but now a widespread agricultural weed throughout the western hemisphere. It has strong vertical stems that can grow to 3–8 feet and covered with densely stellate pubescent. Although velvetleaf is detrimental to many crops if not controlled, it can be used as a potential commercial crop (Kurokawa et al. 2004). The leaves are edible, and the stems are used to make fiber. The plant can also be used for medicinal purposes to treat fever, dysentery, stomachaches, and other problems.

Chloroplast genome is exceptionally conserved in gene content and organization; complete chloroplast genome sequences have been widely used as a source of valuable data for understanding evolutionary biology, which have been used extensively for plant phylogenetic analyses at family/genus/species levels (Sun et al. 2020). So far, the chloroplast genome of *A. theophrasti* has not been reported. Now, we determined the complete chloroplast genome sequence of *A. theophrasti* based on the next-generation sequencing, and the annotated genomic sequence has been deposited into GenBank with the accession number MT991007.

Molecular samples of *A. theophrasti* were collected from Xiao county, Anhui province, China (34°11′39″N, 116°57′13″E). Voucher specimen was stored at the herbarium of Institute of Chinese Materia Medica (CMMI), China Academy of Chinese Medical Sciences with the specimen voucher number is 341322LY0448. Total genomic DNA was isolated using a DNeasy Plant Mini Kit (Qiagen Co., Hilden, Germany). And the sequencing library was constructed and quantified following the methods introduced by Dong et al. (2017). The whole-genome sequencing was conducted with 150 bp paired-end reads on the Illumina HiSeq X Ten platform. Contigs were assembled from the high-quality paired-end reads by using the SPAdes version 3.6.1 program (Kmer = 95) (Bankevich et al. 2012) after filtering low quality reads. The chloroplast genome contigs were selected by the Blast program (Altschul et al. 1990), with the chloroplast genome of *Malva parviflora* (GenBank: MK860036) as the reference. The selected contigs were assembled using Sequencher version 4.10 (Gene Codes Corporation, Ann Arbor, MI, http://www.genecodes.com). Gene annotation was performed with Plann (Daisie et al. 2015) and manually corrected for codons and gene boundaries using the Blast searches.

The complete chloroplast genome reported here is 160,446 bp in length, including two inverted repeats (IRs) of 25,064 bp, which are separated by a large single-copy (LSC) and a small single-copy (SSC) of 89,089 bp and 21,229 bp, respectively. The overall GC-content of the chloroplast genome was 36.9%. The chloroplast DNA of *A. theophrasti* comprised 113 distinct genes, including 79 protein-coding genes, four transfer RNA, and 30 ribosome RNA. In these genes, 17 harbored a single intron, while two (ycf3 and clpP) contained double introns.

We downloaded 22 published chloroplast genomes of Malvaceae and other four chloroplast genomes as outgroup taxa from Genbank to perform the phylogenetic reconstruction. All chloroplast genome sequences were aligned using MAFFT online (Katoh et al. 2019) and ambiguous alignment regions were trimmed by Gblocks (Castresana 2000). The maximum likelihood (ML) analyses were performed in RAxML version 8.1.2432 (Stamatakis 2014). The support branches (BS) were assessed with 1000 rapid bootstrapping replicates. The phylogenetic tree showed that all species of Malvaceae form a monophyletic group with 100% support, and *A. theophrasti* is located in the basal position in Malveae (Figure 1). The chloroplast genome of *A. theophrasti* provided a lot of genetic information for species conservation and identification of Malvaceae.

**Figure 1.**
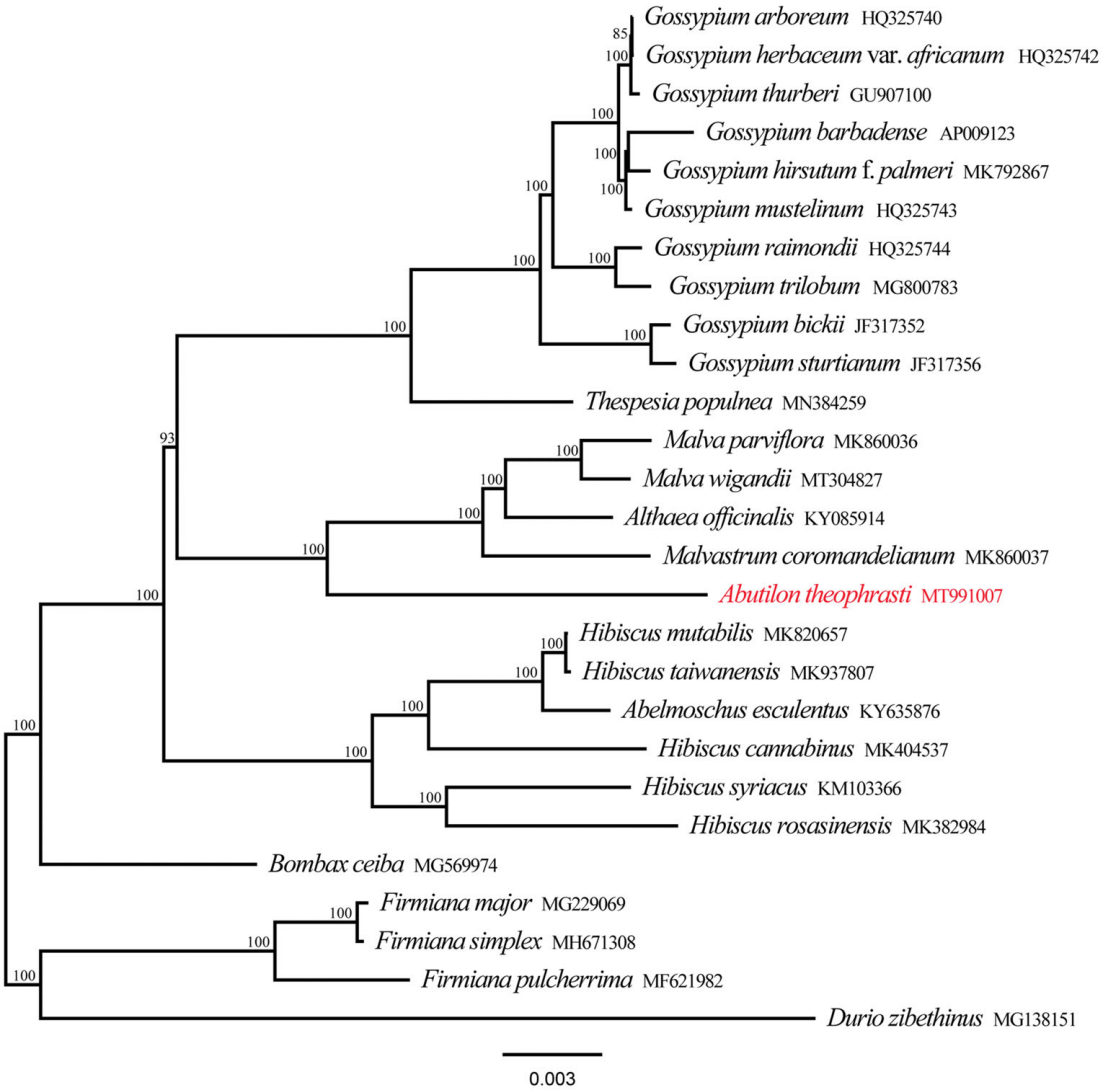
Phylogenetic tree reconstruction of 27 taxa using maximum likelihood (ML) methods in the chloroplast genome sequences. ML bootstrap support value presented at each node.

## Data Availability

The data that support the findings of this study are openly available in GenBank of NCBI https://www.ncbi.nlm.nih.gov/, reference number MT991007, raw data bioproject: PRJNA660005, SRA accession: SRR12649607.
